# Transient Overexposure of Neuregulin 3 during Early Postnatal Development Impacts Selective Behaviors in Adulthood

**DOI:** 10.1371/journal.pone.0104172

**Published:** 2014-08-05

**Authors:** Clare Paterson, Amanda J. Law

**Affiliations:** 1 Department of Psychiatry, University of Colorado, School of Medicine, Aurora, Colorado, United States of America; 2 Department of Cell and Developmental Biology, University of Colorado, School of Medicine, Aurora, Colorado, United States of America; United Graduate School of Child Development, Osaka University, Japan

## Abstract

Neuregulin 3 (NRG3), a specific ligand for ErbB4 and a neuronal-enriched neurotrophin is implicated in the genetic predisposition to a broad spectrum of neurodevelopmental, neurocognitive and neuropsychiatric disorders, including Alzheimer's disease, autism and schizophrenia. Genetic studies in schizophrenia demonstrate that risk variants in NRG3 are associated with cognitive and psychotic symptom severity, accompanied by increased expression of prefrontal cortical NRG3. Despite our expanding knowledge of genetic involvement of NRG3 in neurological disorders, little is known about the neurodevelopmental mechanisms of risk. Here we exploited the fact that a paralog of NRG3, NRG1, readily penetrates the murine blood brain barrier (BBB). In this study we synthesized the bioactive epidermal growth factor (EGF) domain of NRG3, and using previously validated *in-vivo* peripheral injection methodologies in neonatal mice, demonstrate that NRG3 successfully crosses the BBB, where it activates its receptor ErbB4 and downstream Akt signaling at levels of bioactivity comparable to NRG1. To determine the impact of NRG3 overexpression during one critical developmental window, C57BL/6 male mice were subcutaneously injected daily with NRG1-EGF, NRG3-EGF or vehicle from postnatal days 2–10. Mice were tested in adulthood using a comprehensive battery of behavioral tasks relevant to neurocognitive and psychiatric disorders. In agreement with previous studies, developmental overexposure to NRG1 induced multiple non-CNS mediated peripheral effects as well as severely disrupting performance of prepulse inhibition of the startle response. In contrast, NRG3 had no effect on any peripheral measures investigated or sensorimotor gating. Specifically, developmental NRG3 overexposure produced an anxiogenic-like phenotype and deficits in social behavior in adulthood. These results provide primary data to support a role for NRG3 in brain development and function, which appears to be distinct from its paralog NRG1. Furthermore we demonstrate how perturbations in NRG3 expression at distinct developmental stages may contribute to the neurological deficits observed in brain disorders such as schizophrenia and autism.

## Introduction

Convergent evidence suggests that a broad spectrum of neurodevelopmental and psychiatric disorders including, schizophrenia, autism, ADHD and intellectual disability are associated with mutations in genes involved in key neurodevelopmental pathways [1]–[6]. Genome-wide linkage and fine mapping studies demonstrate that structural and polymorphic variation in one such gene, Neuregulin 3 (NRG3), is associated with increased risk for a range of neurodevelopmental disorders including schizophrenia, and atypical neurocognitive and behavioral disorders in humans, including speech delay, delusion severity, attention sustainability and scholastic disorganization [7]–[14]. Furthermore, schizophrenia risk-associated genetic variation in NRG3 impacts human prefrontal cortical physiology during working memory [15]. The same common risk variant in NRG3 is associated with elevated transcriptional levels of NRG3 in both adult and fetal prefrontal cortex (PFC) [13]. Additionally, in agreement with convergent evidence suggesting altered NRG3 expression is pathophysiologically relevant in normal brain function, NRG3 expression is elevated in the PFC of patients with schizophrenia, compared to unaffected individuals [13].

While the evidence for involvement of NRG3 in neurodevelopmental and psychiatric disorders is mounting, little is known about its neurobiological role. NRG3 is a neurotrophic factor, a specific ligand for the receptor tyrosine kinase ErbB4 [16] and a paralog of the growth factor NRG1, all of which are strong candidate risk genes for schizophrenia. Manipulation of NRG1 and ErbB4 in rodents leads to behavioral and neurophysiological phenotypes relevant to schizophrenia, consistent with their known roles in neuronal development, myelination and neurotransmitter function [17]–[19]. Unlike NRG1, expression of NRG3 is limited to the CNS [16], [20], where it is enriched during neurodevelopment [13], [20]. NRG3 promotes oligodendrocyte survival and is implicated in cortical plate development [21], [22]. Despite its structural similarities to NRG1 and disease associations, the neurobehavioral consequences of altered NRG3 signaling are unknown.

Given recent data that demonstrate peripherally administered NRG1 peptides can cross the blood brain barrier (BBB) of neonatal rodents and exert lifelong behavioral and neurochemical effects [23], [24], and observation that NRG3 is pathologically elevated in the brains of patients with schizophrenia [13], we synthesized the bioactive NRG3 EGF peptide and investigated its ability to penetrate the neonatal murine BBB comparative to NRG1. Additionally, we assessed the impact of NRG3 overexposure during early neonatal development (as a peripheral exposure model of NRG3 overexpression in schizophrenia [13]) on a series of adult behaviors relevant to neurocognitive and neurodevelopmental disorders; testing the hypothesis that mice exposed to NRG3 during a critical neurodevelopmental window would show behavioral abnormalities later in life.

Here, we present evidence that like NRG1, NRG3 can readily penetrate the BBB of neonatal mice and is bioactive, inducing activation of the ErbB4-Akt signaling pathway. Moreover, NRG3 overexposure during early neonatal development had life-long consequences on discrete behavioral phenotypes, namely those indicative of anxiety and social development.

Although further studies are required to elucidate the mechanistic role of NRG3 in neurocognitive and neuropsychiatric disorders, the present results provide independent support for 1) previous associations of NRG3 genetic variation to cognitive and behavioral phenotypes in humans; 2) present novel evidence that NRG3 impacts early neonatal brain development where it influences circuitry involved in behaviors related to anxiety and sociability and 3) highlight an experimental rodent model for examining the developmental effects of neurotrophin exposure as it relates to clinical brain disorders.

## Methods and Materials

### Recombinant Neuregulin peptides

A 5 kDa synthetic peptide encompassing the EGF domain of human NRG3 (NRG3-EGF, His286-Asp329; Genbank Accession NP_001010848.2) containing 6 cysteine residues and three disulfide bonds integral to EGF bioactivity [25], [26] was synthesized with an HPLC purity of >95% (AnaSpec Inc., San Jose, CA). BLAST analysis confirmed 100% amino acid conservation of this sequence between human, mouse and rat. As a positive control a synthetic peptide encompassing the EGF domain of human NRG1-β1 (NRG1-EGF, Thr176-Lys246, NP_039250, 8 kDa) was used (R&D Systems, Minneapolis, MN). To study the brain penetrance of peripherally injected NRG synthetic peptides in neonatal brain, an N-terminus biotin conjugated version of the NRG3-EGF peptide was synthesized (AnaSpec Inc.), and the NRG1-EGF peptide (R&D Systems) was biotin labeled using EZ-Link Sulfo-NHS-LC-Biotinylation kit according to the manufacturer's instructions (Pierce, Rockford, IL). All peptides were dissolved in 0.1% bovine serum albumin (BSA)/phosphate buffered saline (PBS) for *in vivo* use.

### Mice and peripheral injection paradigm

All procedures used were in accordance with and approved by the University of Colorado Denver Institutional Animal Care and Use Committee (IACUC), and every effort was made to ameliorate animal suffering. For BBB permeability analysis male neonatal C57BL/6 mouse pups were given a single subcutaneous injection of 1–3 mg/kg NRG3-EGF, 1 mg/kg NRG1-EGF or an equal volume of vehicle (0.1% BSA/PBS) and ErbB4 and AKT phosphorylation determined. From this dose response study 3 mg/kg NRG3-EGF was found to be bioactively equivalent to 1 mg/kg NRG1-EGF, and used in subsequent injection studies. To examine the long term consequences of NRG3 overexposure, male neonatal C57BL/6 mice underwent a sub-chronic injection regimen of a daily subcutaneous injection of 3 mg/kg NRG3-EGF, 1 mg/kg NRG1-EGF or vehicle from postnatal days (PNDs) 2–10 in accordance with previous studies (23, 24).

### Immunohistochemistry

One hour following a single subcutaneous injection of biotin-NRG3-EGF, biotin-NRG1-EGF or vehicle PND2 male mice were euthanized by decapitation and the brain removed. 20βm thick coronal sections were prepared and fixed in 4% paraformaldehyde. The presence of biotinylated NRG3 or NRG1 peptide in these sections was detected using VECTASTAIN Elite ABC kit (Vector Laboratories, Burlingame, CA) followed by incubation with Metal-Enhanced DAB substrate (Pierce) as per the manufacturer's instructions.

### Western blot analysis of NRG3 bioactivity

Three hours following a single subcutaneous injection of NRG3-EGF, NRG1-EGF or vehicle PND2 male mice were euthanized by decapitation and the brain removed. One hemisphere was utilized for western blot analysis as previously described [27], using the following antibodies, phospho-AKT Ser473 (Millipore, Bedford, MA), total-AKT (Cell Signaling Technology, Beverly, MA), phospho-ErbB4 and total-ErbB4 (both Santa Cruz Biotechnology, Santa Cruz, CA). Optical densities of immunoreactive bands were quantified using ImageJ Image Analysis Software (NIH, Bethesda, MD).

### Behavioral analyses

Neonatal male mice were injected daily with 3 mg/kg NRG3-EGF or equal volumes of 0.1% BSA/PBS from PND 2–10. After weaning at PND 21 mice were left undisturbed until behavioral testing at 3–5 months of age. An additional cohort of neonatal mice were injected with 1 mg/kg NRG1-EGF from PND2–10 as previously reported [23], and tested as positive control mice alongside NRG3-EGF exposed mice.

#### General Health

Mice were subjected to a battery of general health measures to assess motoric ability and neurological reflexes as previously described [28].

#### Prepulse Inhibition of the Startle Response

Sensorimotor gating was assessed using three SR-LAB sound attenuated chambers (San Diego Instruments Inc., San Diego, CA), with prepulse amplitudes of 74, 78, 82 and 86 and 90 dB as described previously [28]. % Prepulse inhibition was calculated as follows: 100-(response to prepulse+startle stimulus/response to startle stimulus alone)×100. Startle response *per se* was assessed from the average maximal response to 120 dB startle alone trials.

#### Locomotor Activity Response to a Novel Environment

Open field activity was assessed as distance travelled in a novel open top 42×42×30 cm polyvinyl arena over a 60 minute period, using Ethovision XT video tracking system (Noldus, Leesburg, VA). Time spent and distance travelled in the center portion of the arena was additionally scored.

#### Temporal Order Object Recognition

Mice were assessed for temporal order object recognition memory in the same open field arena used for the locomotor activity task. Temporal order object recognition testing was carried out as previously described [29], [30]. Two sample trials presented 1 hour apart lasted 5 minutes allowing the mice to investigate two sets of dissimilar objects. Three hours after the second sample trial the mice underwent a test trial where time spent investigating objects from each of the sample trials was measured. The discrimination ratio of the test trial is calculated as follows: (time spent sniffing object 1 (most familiar from sample trial 2) - time spent sniffing object 2 (least familiar from sample trial 1))/total time spent sniffing both objects. Intact recency discrimination would therefore result in a discrimination ratio >0.

#### Amphetamine Induced Locomotor Hyperactivity

Locomotor activity tracking was assessed as before, however, after a 10 minute period of habituation to the open field arena mice received a single intraperitoneal injection of D-Amphetamine (3 mg/kg, Sigma Aldrich, St Louis, MO) or saline. Distance travelled was assessed over the subsequent 75 minute period.

#### Social behavior testing

Testing of sociability and preference for social novelty were carried out using the three-chambered protocol as previously described [31], with time spent in each chamber and entries into each chamber assessed using Ethovision XT video tracking software (Noldus). Time spent sniffing novel mouse or novel object (sociability) or time spent sniffing familiar or novel mouse (social preference), were measured with a stop-watch manually by the investigator blind to treatment group.

#### Statistical Analysis

All data is presented as mean ± s.e.m. All statistical comparisons were performed using IBM SPSS statistics package v21. One way ANOVAs were used to analyze the effect of treatment on phosphorylation levels of ErbB4 and Akt, and in measures of general health. Independent samples t-tests were used to analyze the effect of treatment on performance in the temporal order object recognition task between vehicle and NRG3-EGF treated mice. Repeated measures ANOVAs were used to compare data from sensorimotor gating, sociability, and locomotor activity experiments with “prepulse amplitude”, “chamber” or “time” as the within groups repeated measure, respectively. Post-hoc LSD comparisons between groups were performed only when a significant main effect was observed. Statistical significance was interpreted as p values<0.05.

## Results

### Peripherally injected NRG3-EGF penetrates the BBB of neonatal mice

**Figure 1:**
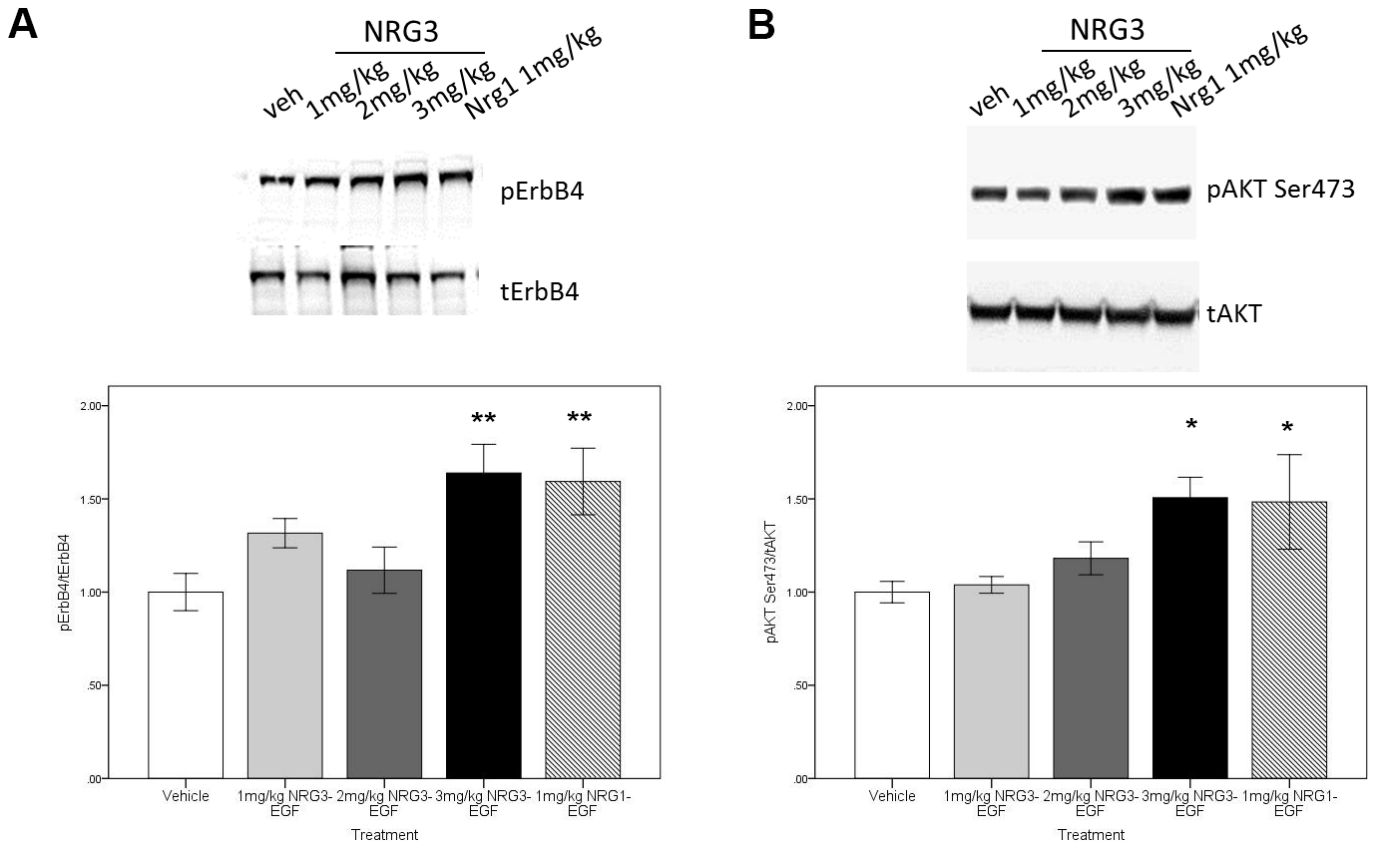
The EGF domain of NRG3 permeates the neonatal mouse BBB in a dose dependent manner, activating ErbB4-Akt signaling. Activation levels of pErbB4 (A), and pAktSer473 (B) in hemi-brain lysates of PND2 mice 3 hours following s.c. injection of vehicle (PBS/0.1% BSA), 1–3 mg/kg NRG3-EGF or 1 mg/kg NRG1-EGF. Representative blots of tErbB4, pErbB4, tAKT and pAKT ser473 protein levels are depicted (upper panels). N = 4–5/treatment group, data represents mean± s.e.m ratio of the respective phosphorylated/total protein expression relative to vehicle treatment group. *p<0.05, **p<0.01 compared to vehicle treated group.

To determine the BBB permeability of NRG3-EGF in the neonatal mouse, and to assess bioactivity of the NRG3-EGF peptide we measured phosphorylation levels of the receptor tyrosine kinase ErbB4 and downstream signaling target Akt following a single subcutaneous injection of NRG3-EGF at doses ranging 1–3 mg/kg compared to that of 1 mg/kg NRG1-EGF. Immunoblotting of whole brain homogenates, at the previously reported maximal activation time-point for NRG1-EGF (3 hours, see [23]), revealed that NRG3-EGF and NRG1-EGF injection induced ErbB4 (Figure 1A) and Akt (Figure 1B) phosphorylation in a dose dependent manner with 3 mg/kg NRG3-EGF eliciting comparable levels of activation as 1 mg/kg NRG1-EGF. These findings indicate that NRG3-EGF can penetrate the neonatal mouse BBB and exert bioactivity comparable to that of NRG1. Total levels of ErbB4 and Akt were unaltered.

Additionally, the penetrance of the NRG3-EGF and NRG1-EGF across the BBB of neonatal mice at a dose of 3 mg/kg or 1 mg/kg, respectively, was confirmed by immunohistochemical analysis of brain sections following injection of biotinylated labeled peptides. Biotin signal was detectable in the prefrontal cortex of mice injected with biotin-NRG1-EGF (Figure S1C) or biotin-NRG3-EGF (Figure S1D), compared to that of the prefrontal cortex of vehicle treated mice (Figure S1B).

### Neonatal NRG3-EGF treatment has no effect on neurodevelopmental milestones or general health

To determine the viability of using the dose of 3 mg/kg NRG3-EGF as a model of overexposure to NRG3 during early postnatal development in mice, we examined the effects of daily subcutaneous injection of 3 mg/kg NRG3-EGF, vehicle or the bioactively comparable dose of 1 mg/kg of NRG1-EGF on neurodevelopmental milestones. NRG3-EGF treatment had no effect on any of the developmental milestones assessed (Table 1). While there was a significant effect of “treatment” on eyelid opening and upper tooth eruption (F(2,26) = 39.78, p<0.001 and F(2,26) = 4.14, p<0.05, respectively) p*ost hoc* analysis revealed this effect was due solely to NRG1-EGF treated mice displaying significantly premature onset of both eyelid opening and upper tooth eruption compared to vehicle and NRG3-EGF treated mice (p<0.05). The onset of lower tooth eruption was unaffected by peripheral treatment (F(2,26) = 0.960, p>0.05). All mice that received daily injections of 3 mg/kg NRG3-EGF survived to adulthood, and were indistinguishable from vehicle treated mice or NRG1-EGF treated mice in measures of general health, motoric ability and neurological reflexes (Table 1).

**Table 1:** Effects of neonatal peripheral overexposure to NRG1 and NRG3 on developmental milestones and general health in adulthood.

	Treatment		
	Vehicle	1 mg/kg NRG1-EGF	3 mg/kg NRG3-EGF
**Phenotype**			
Day of eyelid opening	13.9±0.2	10.6±0.5*** ###	14±0.2
Day of upper tooth eruption	11.7±0.2	10.8±0.4* ^##^	11.9±0.2
Day of lower tooth eruption	10.4±0.5	26.0±1.4	27.9±0.5
**General Health**			
Body weight (g)	29.3±1.08	26.0±1.4	27.9±0.7
Poor coat condition (%)	0	0	0
Bald patches (%)	0	0	0
Missing whiskers (%)	0	0	0
Piloerection (%)	0	0	0
Body tone (% of good)	100	100	100
Limb tone (% of good)	100	100	100
Physical abnormalities (%)	0	0	0
**Motoric abilities**			
Trunk curl (%)	100	100	100
Forepaw reaching (%)	100	100	100
Wire hang (sec)	56.9±3.09	60.0±0	57.45±2.99
Positional passivity (%)	0	0	0
**Reflexes (% of mice normal)**			
Righting reflex (%)	100	100	100
Corneal (%)	100	100	100
Ear twitch (%)	100	100	100
Whisker twitch (%)	100	100	100
**Reactivity**			
To handling (3 point scale)	2.0±0.0	2.0±0.0	2.0±0.0
Petting escape (%)	0	0	0
**Empty cage behavior**			
Transfer freezing (%)	0	0	0
Wild running (%)	0	0	0
Exploration (3 point scale)	2.0±0.0	2.0±0.0	2.0±0.0
Grooming (sec)	12.1±1.64	9.8±4.1	10.55±1.99
Grooming (events)	8.72±2.38	10.0±4.5	6.58±1.45
Rearing (events)	42.27±4.44	42.2±7.2	40.08±2.11

Following daily injections of Vehicle, 1 mg/kg NRG1 or 3 mg/kg NRG3 mice were assayed for neurodevelopmental milestones, including eye opening and tooth eruption, general health, motoric ability and reflexes in adulthood. N = 9–14/treatment group. Data represent mean ± s.e.m.

*p<0.05,

***p<0.001 vs. Vehicle treated mice;

# p<0.05.

### p<0.001 vs. 3 mg/kg NRG3-EGF treated mice.

Moreover, peripheral injections were not associated with any abnormalities of reflexes of ear twitch, corneal reflex, whisker twitch or righting reflex; moreover, motoric abilities of trunk curl, positional passivity, petting escape and wire hand were intact in all mice tested. Peripheral treatment had no overall effect on rearing activity, grooming events or digging events.

These data suggest that despite being administered a relatively high dose of 3 mg/kg NRG3-EGF (which was chosen because it is bioactively comparable to 1 mg/kg NRG1-EGF), gross effects of NRG3 on health and development were not observed, suggesting that the mice were healthy and capable of performing subsequent behavioral analyses.

### Neonatally NRG3-EGF treated mice display elevated anxiety-like behaviors in adulthood

**Figure 2:**
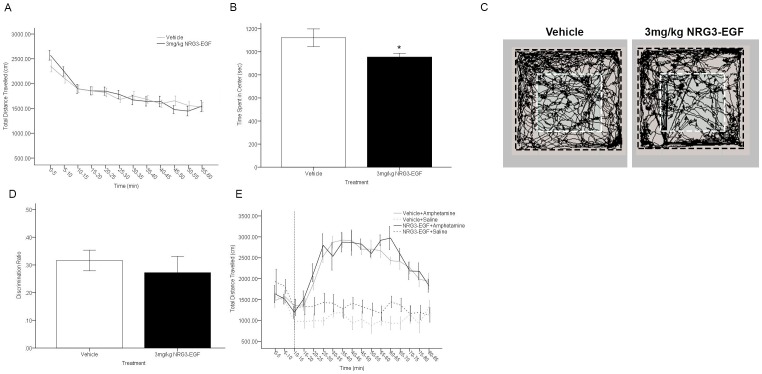
Overexposure to NRG3 during the neonatal period induces an anxiety-like phenotype in adulthood, but has no impact on locomotor activity or temporal order recency discrimination. (A) Total distance travelled in 5 minute intervals over a 1 hr exposure to the open field arena did not differ between NRG3-EGF or Vehicle treated mice (n = 12–14/treatment group). (B)Time spent in the center of the open field over a 1 hr period was significantly reduced in mice treated with NRG3-EGF as neonates compared to control treated mice (n = 12–14/treatment group). (C) Representative track plots of exploration in the open field of vehicle (left panel) and NRG3-EGF (right panel) overexposed mice. Inner area designated by white dashed line designates the center arena. (D) Adult mice treated with NRG3-EGF or vehicle during PND 2–10 performed successfully in the temporal order object recognition task of recency discrimination as adults (Discrimination ratio >0, n = 11–14/treatment group). (E) After a 10 minute period of habituation to the open field (indicated by dashed vertical line), mice neonatally overexposed to NRG3-EGF or vehicle were given a single i.p. injection of saline or 3 mg/kg amphetamine. No effect of treatment (NRG3-EGF) was observed on total distance travelled in 5 minute intervals of the subsequent 75 minutes in the context of amphetamine at a dose of 3 mg/kg (n = 6–7/group). Data represents mean ± s.e.m., *p<0.05 compared to vehicle treated mice.

We tested whether neonatal NRG3-EGF treatment alters baseline locomotor activity in a novel environment in adulthood. Both vehicle and NRG3-EGF treated mice habituated to the open field arena over the 60-minute task as indicated by a significant main effect of time on distance travelled (F(11,264) = 17.961, p<0.001). General locomotor activity in the open field was unaffected by neonatal NRG3 overexposure (treatment = F(1,24) = 0.011, p = >0.05, treatment×time interaction = F(11,264) = 1.412,p = >0.05)(Figure 2A). However, time spent in the center of the open field arena during the 60-minute task was significantly reduced in mice treated with 3 mg/kg NRG3 (t(24) = 2.103, p<0.05) (Figure 2B). These alterations in exploratory behavior in the open field following NRG3-EGF overexposure are apparent when investigating the track plots of exploratory behavior, where mice treated with NRG3-EGF show a tendency to remain in the periphery of the open field arena, crossing the center arena less frequently than vehicle treated mice (Figure 2C).

### Neonatal overexposure to NRG3-EGF does not disrupt temporal order recency discrimination memory or amphetamine-induced hyperlocomotor activity

In order to investigate the effects of increased NRG3 signaling during early brain development on cognitive behavioral tasks in adulthood we examined the effect of neonatal NRG3-EGF overexposure on performance in the temporal order object recognition task of recency discrimination memory. This task depends upon fully functioning prelimbic cortical and hippocampal circuitry to discriminate between objects of differing familiarity [29], [30]. Neonatal over-exposure to NRG3 did not impact temporal order recognition memory (t(24) = 0.608, p>0.05), with both vehicle and NRG3-EGF treated mice showing a discrimination ratio >0 (Figure 2D). To determine if peripheral overexposure to NRG3-EGF during early development altered motoric responses to amphetamine in adulthood, we compared the distance travelled in the open field in response to a single intraperitoneal single injection of amphetamine or saline in mice treated with NRG3-EGF or vehicle during PNDs2–10. 3 mg/kg amphetamine treatment significantly increased locomotor activity in the open field F(1,21) = 34.02, p<0.001; however there was no significant effect of peripheral treatment (NRG3-EGF vs. Vehicle; F(1,21) = 2.105, p>0.05) or interaction between peripheral treatment and amphetamine treatment at this individual dose investigated(F(1,21) = 0.175, p>0.05) on locomotor activity (Figure 2E).

### Neonatal overexposure to NRG1-EGF but not NRG3-EGF disrupts sensorimotor gating

**Figure 3:**
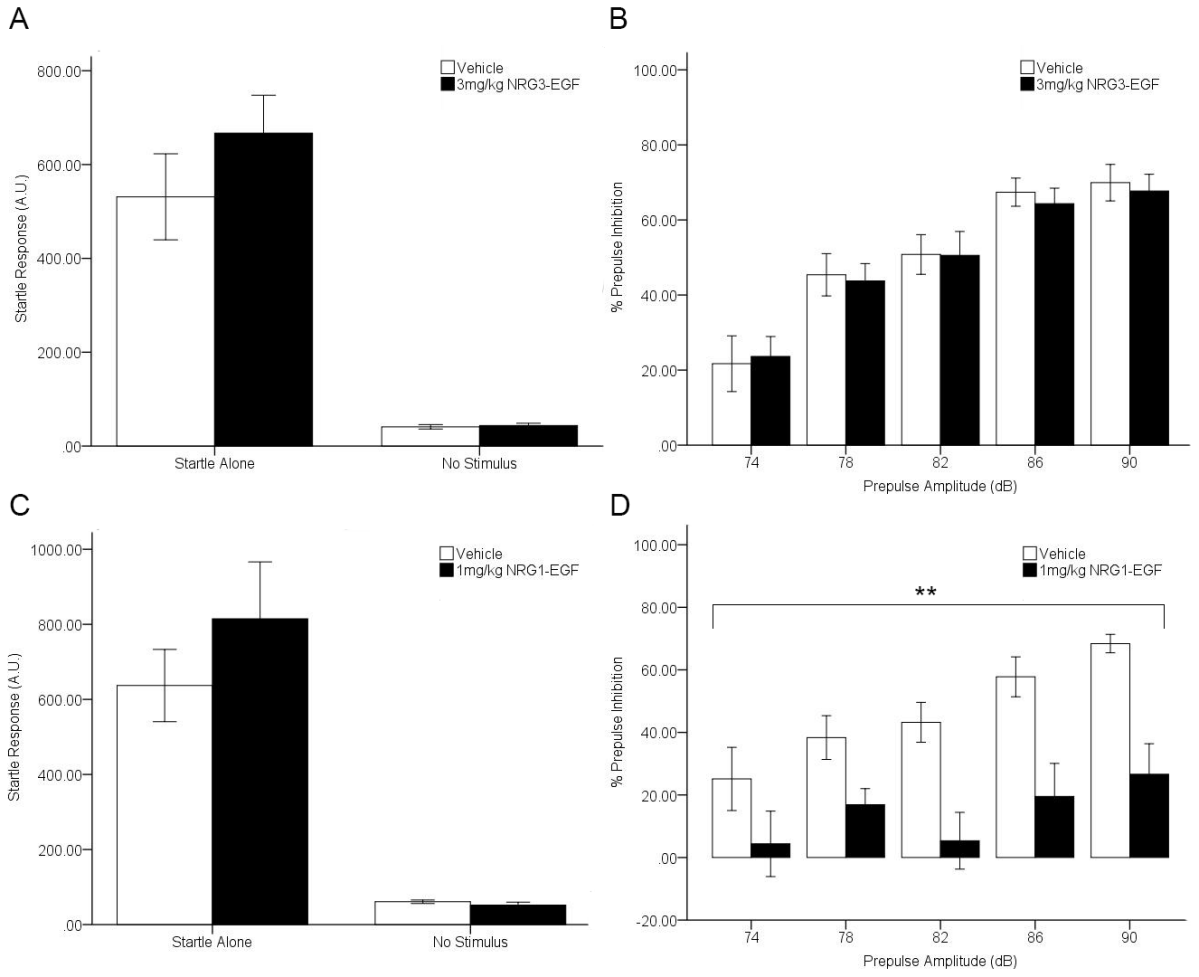
Neonatal overexposure to NRG1 but not NRG3 impairs sensorimotor gating in adulthood. The effect of daily treatment from PND 2–10 with 3 mg/kg NRG3-EGF (A, B) or 1 mg/kg NRG1-EGF (C, D) on startle responses to the presentation of no stimulus or a 120 dB stimulus (A, C) and % prepulse inhibition of the startle response to the presentation of prepulse stimuli at amplitudes of 74, 78, 82, 86 and 90 dB (B, D) in adulthood. Data represents mean ± s.e.m., n = 8–14/treatment group. **p<0.01, main effect of treatment.

Mice treated with NRG3-EGF, NRG1-EGF or vehicle during PND2–10 were tested at adulthood for prepulse inhibition of the startle response as a measure of sensorimotor gating. NRG3 treatment had no significant effect on the level of response to startle-alone (t(24) = −1.117, p>0.05) or no stimulus trials (t(24) = −0.425, p>0.05) which allows for unhindered interpretation of prepulse inhibition results (Figure 3A). Both vehicle and NRG3-EGF treated mice display prototypical sensorimotor gating ability, exhibiting increased levels of prepulse inhibition with increased prepulse amplitude as demonstrated by a significant main effect of prepulse amplitude (F(4,96) = 42.37, p = <0.001). No main effect of NRG3 treatment was observed on prepulse inhibition (F(1, 24) = 0.033, p>0.05), nor a significant interaction between NRG3 treatment and prepulse amplitude (F(4,96) = 0.121, p>0.05) (Figure 3B). NRG1-EGF treatment had no effect on startle-alone response (t(15) = −1.016, p = >0.05)) or basal activity during no stimulus trials (t(15) = 1.002, p>0.05) (Figure 3C), however, there was a significant main effect of NRG1 treatment on %PPI (F(1,15) = 13.15, p<0.01), with NRG1-EGF treated mice displaying stark deficits in sensorimotor gating at all prepulse amplitudes (p<0.05)(Figure 3D).

### Neonatal NRG3-EGF treatment alters adult social behaviors

**Figure 4:**
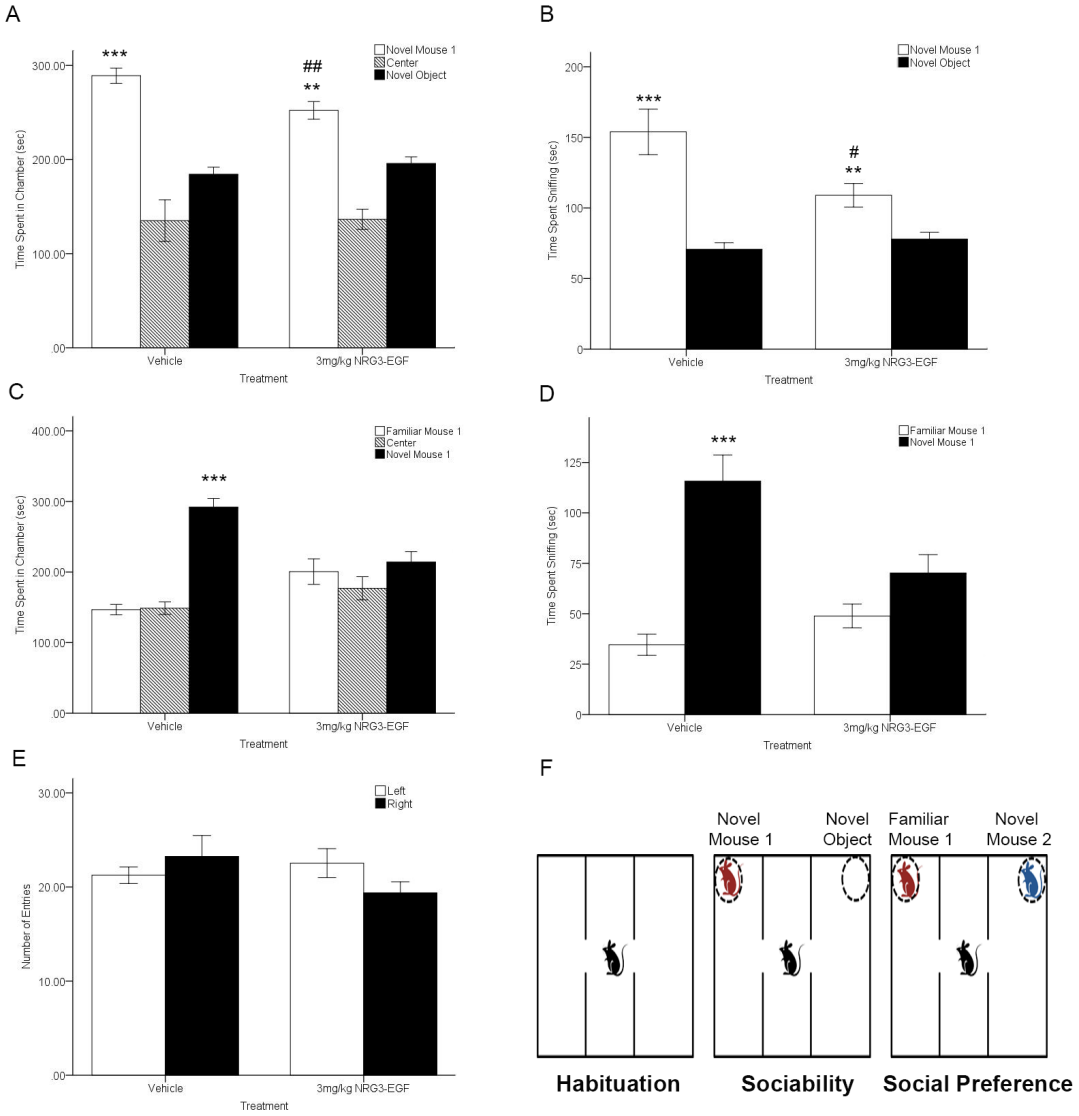
Neonatal overexposure to NRG3 impairs social behaviors in adulthood. (A) Vehicle and NRG3-EGF treated mice spent more time in chamber with the novel mouse 1 than a novel object in the 3-chamber test of sociability. (B) Both treatment groups showed preference for the novel mouse 1 in time spent sniffing; however, the preference was blunted in mice overexposed to NRG3-EGF compared to vehicle treated mice. (C) NRG3-EGF treated mice showed absence of preference for social novelty, spending equal time in the chamber with familiar mouse 1 and novel mouse 2. (D) Sniff time at familiar mouse 1 and novel mouse 2 was comparable in NRG3-EGF overexposed mice, whereas vehicle treated mice showed a significant preference for the more unfamiliar novel mouse 2. (E) During the initial 10-minute habituation period to the apparatus the number of entries was equal between right and left chambers. (F) Schematic showing the setup of the trials in the three-chambered social test apparatus. n = 11–12/treatment group. Data represents mean ± s.e.m. **p<0.01, ***p<0.001 within group comparison (novel mouse 1 vs. novel object or familiar mouse 1 vs. novel mouse 2), #p<0.05,##p<0.01 between group comparison (Vehicle vs. NRG3-EGF treated mice).

Sociability, defined as spending more time in the chamber with a novel mouse (i.e. novel mouse 1) than in the chamber with a novel object, was observed for both vehicle and NRG3-EGF treated mice as demonstrated by a main significant effect of chamber (Sniff time; F(1,23) = 48.72, p<0.001 and time in chamber F(1,22) = 49.054,p<0.001, respectively). However, NRG3-EGF treated mice displayed reduced sociability compared to vehicle treated mice whereby a significant interaction between treatment and chamber was observed for measures of time in chamber (Figure 4A) (F(1,22) = 3.299, p<0.05) and sniff time (Figure 4B) (F(1,23) = 10.182, p<0.01). While neonatal overexposure to NRG3-EGF did not completely ablate sociability, NRG3 exposed mice spent less time sniffing or less time in the same chamber with a novel mouse compared to vehicle treated mice (t(23) = 2.525, p<0.05 and t(23) = 2.967,p<0.001, respectively).

Significant deficits in social novelty preference were observed in NRG3 treated mice, whereby preference for social novelty is defined as propensity to spend time with a novel mouse (novel mouse 2) than with a familiar conspecific (familiar mouse 1). A significant interaction of treatment and chamber was observed (F(1,22) = 7.823, p<0.01 and F(1,23) = 10.337, p<0.01) for time spent in chamber and sniff time, respectively, whereby vehicle treated mice spend more time in the chamber containing the novel mouse (t(23) = 60.9, p<0.001) and spend more time sniffing the novel mouse compared to the familiar mouse (t(23) = 39.28, p<0.001). NRG3-EGF treated mice did not distinguish between the novel and familiar mouse, spending equal amounts of time in each chamber (Figure 4C) (t(23) = 2.08, p>0.05) and equal time sniffing both mice (Figure 4D) (t(23) = 2.575, p>0.05). During the habituation phase, prior to social novelty and social preference testing there was no preferential chamber side (chamber F(1,23) = 0.127, p>0.05) or effect of treatment on the number of entries into either chamber side (treatment F(1,23) = 0.809, p>0.05) (Figure 4E). Together, these data indicate that social development is impaired in the context of NRG3 neonatal overexposure.

## Discussion

Here we provide novel evidence, that peripherally injected NRG3 can cross the BBB of neonatal mice, and that overexposure to NRG3 during early postnatal life leads to alterations in anxiety-like and social behaviors in adulthood. These data demonstrate the relevance of NRG3 in normal brain development and function, and provide initial insight into how altered NRG3 signaling may be pathophysiologically relevant to neuropsychiatric and neurodevelopmental disorders.

The impermeability of the BBB to peripheral peptides has limited the design of many therapeutics and has also hindered the study of many proteins relevant to neurobiological illnesses [32], therefore it is remarkable that NRG3 successfully penetrates the BBB. NRG1 and EGF can permeate from the periphery to the brain of neonatal and adult rodents utilizing the same injection paradigm as described herein [23], [33], [34]. Although the mechanism of how NRG1 crosses the BBB is not fully understood, it is thought to be receptor mediated involving ErbB3 and ErbB4 [35]. Given that NRG3 selectively binds to ErbB4 receptors [16], it is likely that NRG3 uses similar mechanism of transport to penetrate the BBB. In the current study we demonstrate that peripheral NRG3 treatment can activate brain ErbB4-Akt signaling to the same extent as NRG1, confirming the bioactivity of our NRG3-EGF peptide and the validity of our model system approach. Our findings that a higher dose of NRG3-EGF than NRG1-EGF was required to induce a comparable level of ErbB4-Akt signaling activation is consistent with previous reports that NRG3 has a lower binding affinity to ErbB4 than NRG1 [36]. Additionally, NRG1 (unlike NRG3) can also initiate Akt signaling via ErbB2 and ErbB3 receptor activation [36].

The dose of 3 mg/kg NRG3 had no effects on mortality, weight, neurodevelopmental milestones or general health measures. However, in agreement with previous studies of neonatal overexposure to NRG1 and other epidermal growth factors, [23], [33], [37] we identified that NRG1 exposure stimulates precocious eye opening and tooth eruption. These results suggest while 3-fold higher than NRG1, the dose of NRG3-EGF given was well tolerated and subsequent behavioral changes were CNS driven rather than a peripheral effect. The peripheral effects observed in neonatally administered NRG1 mice is likely due to NRG1's widespread expression and function in multiple organ systems including the heart, breast and nervous system [38]. Conversely, NRG3 expression is neuronally enriched and therefore its actions are likely more restricted to the brain. Moreover, the EGF domains of NRG1 and NRG3 share only 31% sequence homology and therefore it is probable some of their biological actions are distinct.

Non-redundancy between the growth factors is further evidenced by our findings that the behavioral consequences of neonatal overexposure to NRG3 are non-overlapping with that of overexposure to NRG1. For example, while NRG1 overexposure in neonates has been previously demonstrated to induce increased sensitivity to the locomotor effects of amphetamine [23], we present evidence that at 3 mg/kg amphetamine induced locomotor activity is enhanced to the same extent in vehicle and NRG3-EGF overexposed mice. However, since only a single relatively high dose of amphetamine was tested in the current study, future investigations of sensitivity to lower doses of amphetamine are required to rule out the possibility of a ceiling effect. Importantly we present evidence for neonatal overexposure to NRG1 inducing sensorimotor gating deficits in adulthood in agreement with previous data [23]. PPI deficits as well as memory deficits are also reported in several transgenic NRG1 mouse lines [17], [39]–[41] as well as neonatal overexposure to EGF [42]. Additionally, the finding of a long-term behavioral consequence of neonatal NRG1 over-exposure is convergent with previous reports and validates our use of this neonatal growth factor based peripheral injection paradigm.

NRG3 overexposure during the neonatal period had lifelong effects on anxiety in adulthood as demonstrated by increased time spent in the center of the open field arena. This suggests that NRG3 is critical in the expansion of neurocircuitry involved in anxiogenesis during early postnatal development, however, further studies employing more sophisticated measures of anxiety such as elevated plus maze are warranted. Consistent with these observations, mice with decreased ErbB4 signaling (an opposing model to that described in the current study) show reduced anxiety levels in adulthood [19], [43] and mice hypomorphic for NRG3 are less anxious in response to nicotine withdrawal [44]. While anxiety was not assessed in adult mice neonatally overexposed to NRG1, peripheral exposure to NRG1 in adult mice induces an anxiolytic phenotype [45], therefore, it is possible that NRG3 may also have temporally regulated effects. It is likely the neurotrophic actions of neuregulins are highly dependent upon the developmental stage of exposure, the duration of exposure and which cell types the growth factors act upon. NRG3 specifically binds to and activates ErbB4 receptors, which are exclusively expressed on inhibitory interneurons where they regulate synaptic plasticity [43], [46].

Consistent with overexposure to NRG1 during early development [23], peripheral injection of NRG3 in neonates led to social deficits in adulthood. During early neonatal development, serotonin (5-HT) plays a fundamental role in modulating anxiety and social behaviors [47]. While neurotransmitter levels were not assessed in the present study, it is probable that NRG3 is important in regulating 5-HT and dopaminergic neurotransmission as has been previously demonstrated for NRG1 [23], [48]. Additionally, 5-HT and dopamine are also important regulators of impulse control which is influenced by NRG3 expression levels in mice [49].

The circuitry that drives social behaviors appears to be particularly sensitive to manipulation during early postnatal life with many studies demonstrating that aberrations during this critical period are linked to long lasting deficits in social capabilities [50]. During the early postnatal period in mice, multiple critical processes are underway including axonal elaboration and synaptogenesis [51], as well as structural reorganization of the developing neocortex such as cortical plate positioning [52]. Interestingly, NRG3 expression is enriched in the cortical antihem during late embryogenesis, where it is thought to be important in the regulation of cortical patterning [21]. Therefore, it is possible that transient overexposure to NRG3 during early postnatal life disrupts cortical circuit development which is critical for normal mature brain function. Other neurodevelopmental models studying early developmental factors that may increase predisposition to schizophrenia have highlighted the importance of the neonatal period in the pathogenesis of schizophrenia and other neuropsychiatric disorders including the neonatal lesion model in rodents [53] and non-human primates [54] which elicit a broad spectrum of schizophrenia related behavioral phenotypes.

Finally, although further work is needed to determine the biological role of NRG3 at differing critical periods of development, the current study bridges a gap in understanding the pathophysiological role of NRG3 in neurodevelopmental disorders such as schizophrenia and autism, and provides a valuable rodent model system for study of developmental neurotrophin overexposure without need for genetic manipulation. Together, our data suggest that NRG3 plays a prominent role in early postnatal brain development where it potentially modulates the construction and plasticity of newly developing brain circuits relevant to anxiety and social cognition.

## Supporting Information

Figure S1 **Immunohistochemical evidence for the penetrance of NRG3-EGF and NRG1-EGF across the BBB of neonatal mice.** Schematic showing the location of the mouse brain examined for the presence of biotinylated peptide (adapted from the Allen Developing Mouse Brain Atlas, Allen Institute for Brain Science. Available at: http://mouse.brain-map.org), rectangle indicates brain area magnified in images B–D (A). Prefrontal cortical section of PND2 mice stained for the presence of biotin 1 hour following injection of vehicle (B), biotin-NRG1-EGF (C), or biotin-NRG3-EGF (D).
(TIF)
