# Supplementary figures and images for: Transient Overexposure of Neuregulin 3 during Early Postnatal Development Impacts Selective Behaviors in Adulthood
Source: PLoS One. 2014 Aug 5;9(8):e104172. doi: 10.1371/journal.pone.0104172 (PMC4122441; doi:10.1371/journal.pone.0104172)

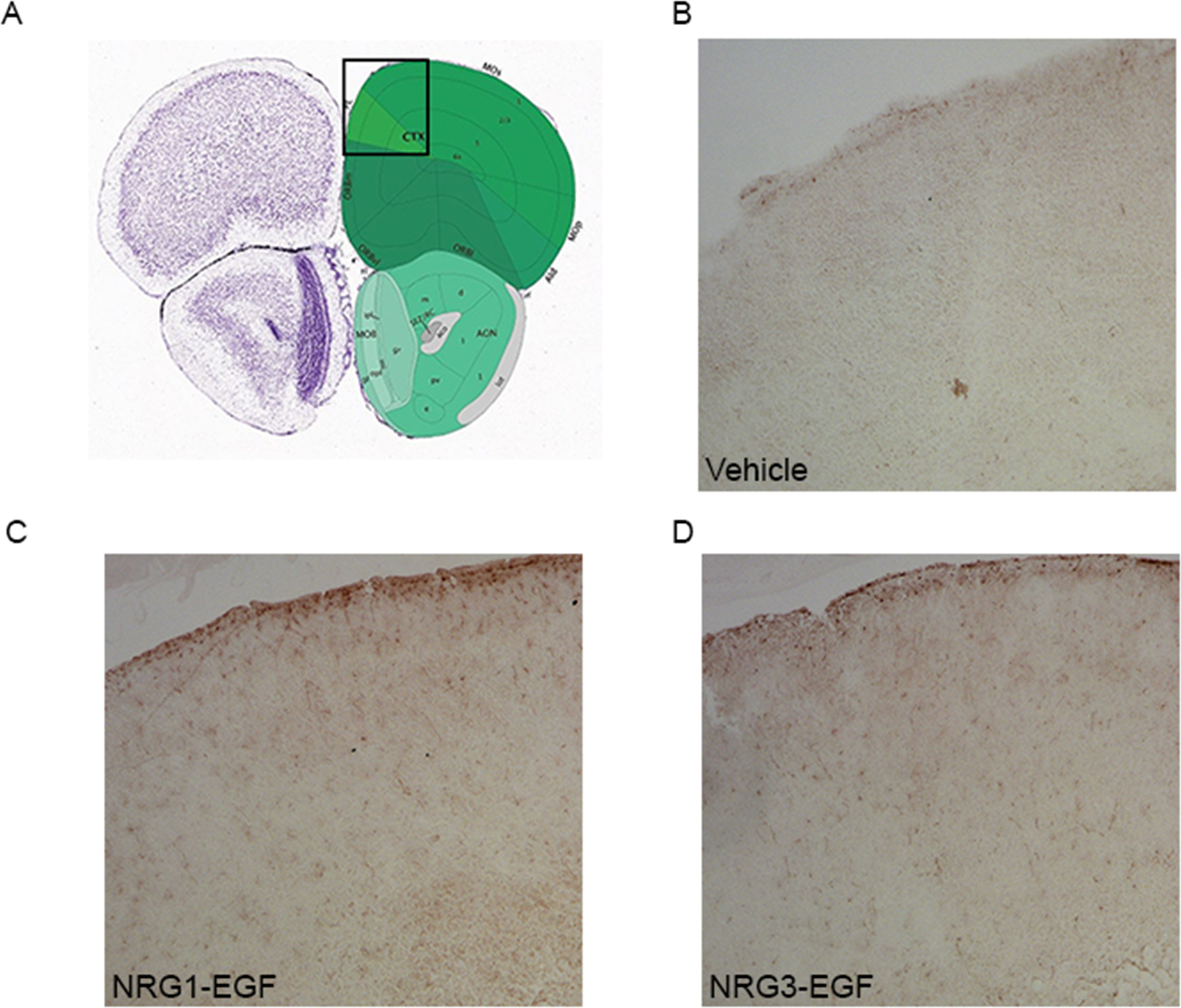

Supplement: Figure S1 — Immunohistochemical evidence for the penetrance of NRG3-EGF and NRG1-EGF across the BBB of neonatal mice. Schematic showing the location of the mouse brain examined for the presence of biotinylated peptide (adapted from the Allen Developing Mouse Brain Atlas, Allen Institute for Brain Science. Available at: http://mouse.brain-map.org), rectangle indicates brain area magnified in images B–D (A). Prefrontal cortical section of PND2 mice stained for the presence of biotin 1 hour following injection of vehicle (B), biotin-NRG1-EGF (C), or biotin-NRG3-EGF (D). (TIF) [file pone.0104172.s001.tif]
